# Encapsulated VEGF_121_-PLA microparticles promote angiogenesis in human endometrium stromal cells

**DOI:** 10.1186/s43141-021-00118-1

**Published:** 2021-02-01

**Authors:** Sunil Abraham, Geetha Sanjay, Noushin Abdul Majiyd, Amutha Chinnaiah

**Affiliations:** 1 Department of Animal Behavior & Physiology, School of Biological Sciences, Madurai Kamaraj University, Madurai, 625021 India; 2 Innov4Sight Health and Biomedical Systems Pvt. Ltd. Biologics Lab- # EGF11, Bangalore Bioinnovation Centre, Bangalore Helix Biotech Park, Electronics City Phase 1, Bangalore, Karnataka 560100 India; 3 CRAFT Hospital and Research Centre, Centre for Excellence in Infertility Treatment, Kodungalur P O, Thrissur, Kerala 680664 India

**Keywords:** Angiogenesis, Endometrium, Human endometrium stromal cells, Migration, Proliferation, VEGF

## Abstract

**Background:**

In this study, Vascular Endothelial Growth Factor 121 expressed abundantly in endometrial stromal cells is encapsulated with poly-l-lactide and characterized the properties for endometrial angiogenesis. We studied the migration, proliferation and the protein levels of human immortalized endometrium stromal cells after treating the cells with recombinant Vascular Endothelial Growth Factor (200 and 500 nanogram), and poly-l-lactide loaded Vascular Endothelial Growth Factor 121 (day 1, 20 and 30). The present study explains endometrium angiogenesis because endometrium plays an important role in pregnancy.

**Results:**

Migration and proliferation studies in endometrium cells proved the efficiency of Vascular Endothelial Growth Factor and poly-l-lactide loaded Vascular Endothelial Growth Factor 121. This proliferated and increased the migration of the cells in vitro and also activated the Protein kinase B, Phosphatidylinositol-4, 5-Bisphosphate 3-Kinase Catalytic Subunit Beta, α-Smooth muscle actin and vascular endothelial growth factor receptor 2 pathways. Western blot analysis showed the increased expression levels of kinases, smooth muscle actin and vascular endothelial growth factor receptor 2 after the treatment with Vascular Endothelial Growth Factor and poly-l-lactide loaded Vascular Endothelial Growth Factor 121 particles in comparison to the control group. The elevated levels of α-Smooth muscle actin in endometrium cells with Vascular Endothelial Growth Factor prove the regulation of angiogenesis in vitro.

**Conclusion:**

Endometrium thickness is one of the important factors during implantation of embryo and pregnancy. Slow release of VEGF from PLA encapsulated microparticle further controls the endothelial cell proliferation and migration and helps in the promotion of angiogenesis. The combined effect studied in vitro could be used as a pro-angiogenic drug on further in vivo confirmation.

## Background

The inner lining of the uterus is called endometrium, and it supports and nourishes the developing baby. The lining is shed if the fertilized egg is not implanted and leads to menstruation which is also called a period. During every menstrual cycle, a new vascular system develops in the endometrium through the process called angiogenesis which supports cell growth and differentiation [1].

The functional layer of the endometrium is differentiated into the glandular epithelium, luminal epithelium, stroma, and vascular compartments [2]. Stromal cells are connective tissue consisting of extracellular matrix (ECM), natural killer (NK) cells, and also spiral arteries for the formation of blood vessels [2–4]. Stromal cells undergo decidualization in the presence of estrogen and progesterone [2].

Vascular endothelial growth factor (VEGF) is an important growth which stimulates proliferation and migration [5] and plays a major role in endometrium repair. There are three phases in the uterine cycle, i.e., menstrual phase, from days 1 to 5—during this period, the endometrial lining undergoes rapid degeneration and regeneration [6, 7]; next comes the proliferative phase, from days 5 to 14—during this period the thickening of the mucosa occurs. During this maturation period, the rising level of estrogen initiates the formation of a new layer and thickens the endometrium [8–10]. The secretory phase of the uterine cycle is from days 14 to 28; during this phase, ovulation occurs and the progesterone is produced by the corpus luteum which plays an important role in the receptivity of endometrium leading to implantation, increases blood flow, reduces contractions in the smooth muscles in the uterus, and supports pregnancy [10, 11].

Endometrium plays an important role in pregnancy; if the thickness of the endometrium is below 7 mm, then it is considered as thin endometrium. In this case, the embryo does not implant leading to implantation failure and menstruation begins. Keeping this preview in mind, the present study is to understand the role of endometrium angiogenesis in vitro.

**Fig. 1:**
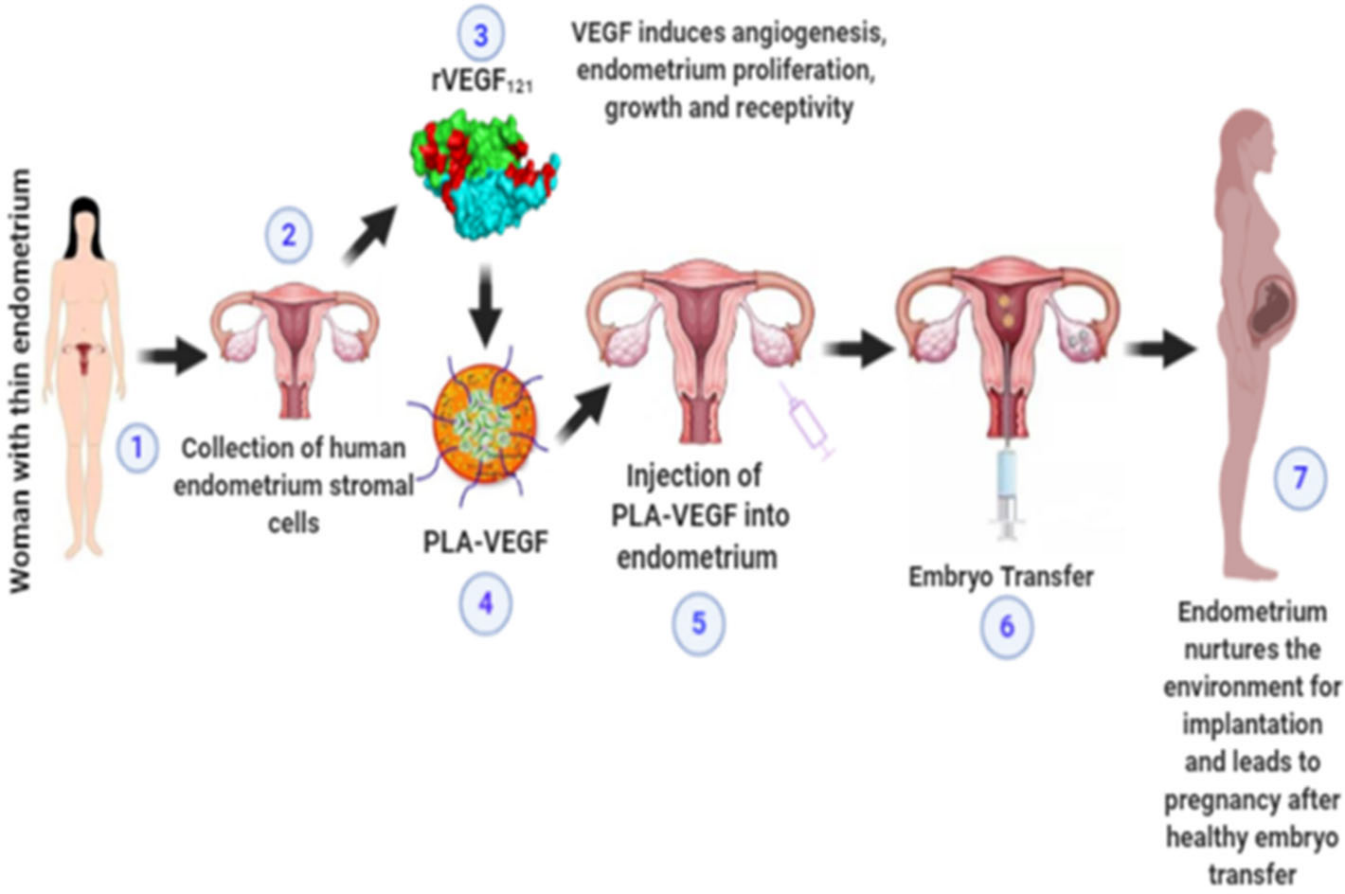
Diagrammatic representation of endometrium regeneration using PLA-VEGF: (1) A woman who is not able to conceive with thin endometrium condition. (2) Collection of the endometrium stromal cells (hESC) from the patient’s endometrium tissues via. hysterectomy. (3) Production of recombinant VEGF_121_. (4) Encapsulation of rVEGF_121_ with poly-l-lactide. (5) Injection of the PLA-VEGF_121_ into the thin endometrium (uterine lining) with the help of IUI catheter. (6) After successful regeneration of the endometrium, embryo which was prepared; transferred in the uterine. (7) After the successful transfer of the healthy embryo, the woman gets pregnant. Successful implantation lies in a fertile environment (i.e., endometrium). In regenerating the endometrium, there are high chances of successful implantation and pregnancy

VEGF_121_, the shortest isoform of VEGF-A, is abundantly expressed in the mid-late proliferative phase of human endometrium stromal cells [12]. Recombinant VEGF_121_ and its microparticles were already characterized in our earlier studies [12]. This 16 kDa protein is more angiogenic than other isoforms, mainly because it can freely diffuse from the cells producing it [13]. On appropriate administration, VEGF can promote angiogenesis and signal vascular endothelial cells which help in cell proliferation and migration, but the therapeutic effects of this protein at high doses is uncertain which could potentially lead to the development of a vascular tumor. Thus, there is a need for a controlled delivery mechanism that will release the bioactive molecules in a sustained mode to the delivery site. Analysis and characterization of rVEGF_121_ and PLA-VEGF were already reported in our earlier publication [12]. Here we have studied the role of VEGF_121_- and PLA-VEGF-mediated signaling for its angiogenesis effect in vitro especially in endometrial stromal cells. Recombinant VEGF_121_- and VEGF_121_-loaded microparticles in different concentrations were treated in immortalized human endometrium stromal cell lines (HESC) and tested for its proliferatory and migratory potential. A typical diagrammatic representation of how VEGF loaded with PLA microparticles can possibly help endometrium regeneration is presented in Fig. 1.

## Methods

### In vitro proliferation assay

Immortalized human endometrium stromal cell lines (HESC) were purchased from ABM, Inc. Canada, and were grown in Prigrow IV medium containing 10% FBS (ABM, Inc. Canada), supplied with 5% CO_2_. Before beginning the experiment, the cells were tested for the presence of mycoplasma and confirmed negative. HESC (5000 cells/well) were cultivated in 96-well plates (Corning, NY, USA) consecutively, in the specified medium with growth factors, in 5% CO_2_ at 37 °C incubator for the cells to adhere to the wells. The medium was replaced with fresh serum and growth factor reduced medium with rVEGF_121_ (200 and 500 ng) and VEGF-loaded PLA microparticles (days 1, 20, and 30) [12] and determined its effects on proliferation. MTT assay was performed as per the manufacturer’s protocol (HiMedia, Mumbai, India). The plate was measured spectrophotometrically at 570 nm with ELISA plate reader (Spectramax M5e, Molecular Devices).

### In vitro migration assay

Twenty-four-well plates (Corning, NY, USA) were seeded with 10,000 HESC cells/wells and grown until they were confluent. The monolayer was scratched with a sterile tip and the cellular debris was washed with PBS. The cells were thereafter incubated with VEGF (study group) and without rVEGF_121_ (200 and 500 ng) (control group) and VEGF-loaded PLA microparticles (days 1, 20, and 30) [12] in serum-free DMEM for at least 48 h for the migration of the cells to the scratched area. The plates were imaged in a camera connected with an inverted microscope (Leica). Image J was used to determine wound healing percentages.

### SDS-PAGE and Western blotting

The HESC cells used for migration assay with respective stimulant, rVEGF_121_ (200 and 500 ng) and VEGF-loaded PLA microparticles (day 1, 20 and 30) after their complete healing were lysed with radio-immunoprecipitation assay buffer (RIPA) (Thermo Fischer Scientific, USA). Briefly, 200 μL of RIPA lysis buffer was added to each well and collected the lysed cells and stored in − 80 °C until used in Western blot.

The harvested proteins with proper controls were analyzed by 12% Sodium Dodecyl Sulphate Polyacrylamide Gel Electrophoresis (SDS-PAGE) under denaturing conditions. Initially, the proteins were estimated by Bicinchoninic acid assay (BCA) protein assay kit (Boster Biological Technology, CA, USA). Ten micrograms of the supernatant (cell lysate) was mixed with 5x SDS-PAGE gel loading buffer, and the samples were boiled at 100 °C for 10 min and the gel was loaded. The gel was run for 2–3 h at 100 Volts and was transblotted onto a Polyvinylidene fluoride (PVDF) membrane using the Trans-Blot Turbo Transfer System (Bio-Rad). Initially, the PVDF membrane (Bio-Rad) was activated with methanol for 90 s as per the manufacturer’s instructions. The transferred blot was blocked with 5% Bovine Serum Albumin (BSA) (Sigma-Aldrich, USA) in 1x Tris Buffered Saline-Tween 20 (TBST) overnight at 4 °C. The blot was detected by incubation with antibody to Protein Kinase B Alpha (PKBa) (Cloud Clone Corp., USA) at a dilution of 1:2000, Phosphoinositide-3-Kinase Catalytic Beta Polypeptide (PIK3Cb) (Cloud Clone Corp., USA) at a dilution of 1:2000, VEGFR2 (Cloud Clone Corp., USA) at a dilution of 1:2000, anti-alpha smooth muscle actin (#ab32575, Abcam, UK) at a dilution of 1:2000; GAPDH loading control antibody (#MA5-15738, Thermo Fischer Scientific, USA) at a dilution of 1:5000. The blot was incubated for 2 h in the primary antibody at room temperature in Stuart Gyro rocker, SSL3 (Cole-Parmer, UK) at a speed of 15 rpm. The blot was further washed with 1x TBST for 6 times, 5 min each in Gyro rocker at a speed of 50 rpm, and incubated with specific secondary antibody, anti-mouse (1:5000)/anti-rabbit (1:10,000) for 1 h in Gyro rocker at 15 rpm. The blot was again washed with 1x TBST for 6 times, 5 min each. The blot was developed using Clarity Western enhanced chemiluminescence (ECL) substrate (Bio-Rad); the images were captured in GE Amersham Imager 600.

### Statistical analysis

Data represented as the mean value of ± SEM from a minimum of three experiments and were analyzed by one-way ANOVA, two-way ANOVA, and Bonferroni post-test with GraphPad Prism—data analysis and graphical software. To normalize the gene expression values, the Mann-Whitney *U* test was performed to compare the two groups. Probability (*P*) value was defined as *p* < 0.05 considered to be statistically significant which is indicated with asterisks. Densitometry analysis was done to normalize the gene expression with Image J software.

## Results

### The enhanced proliferative effect of VEGF and its loaded particle in human endometrium stromal cells

**Fig. 2:**
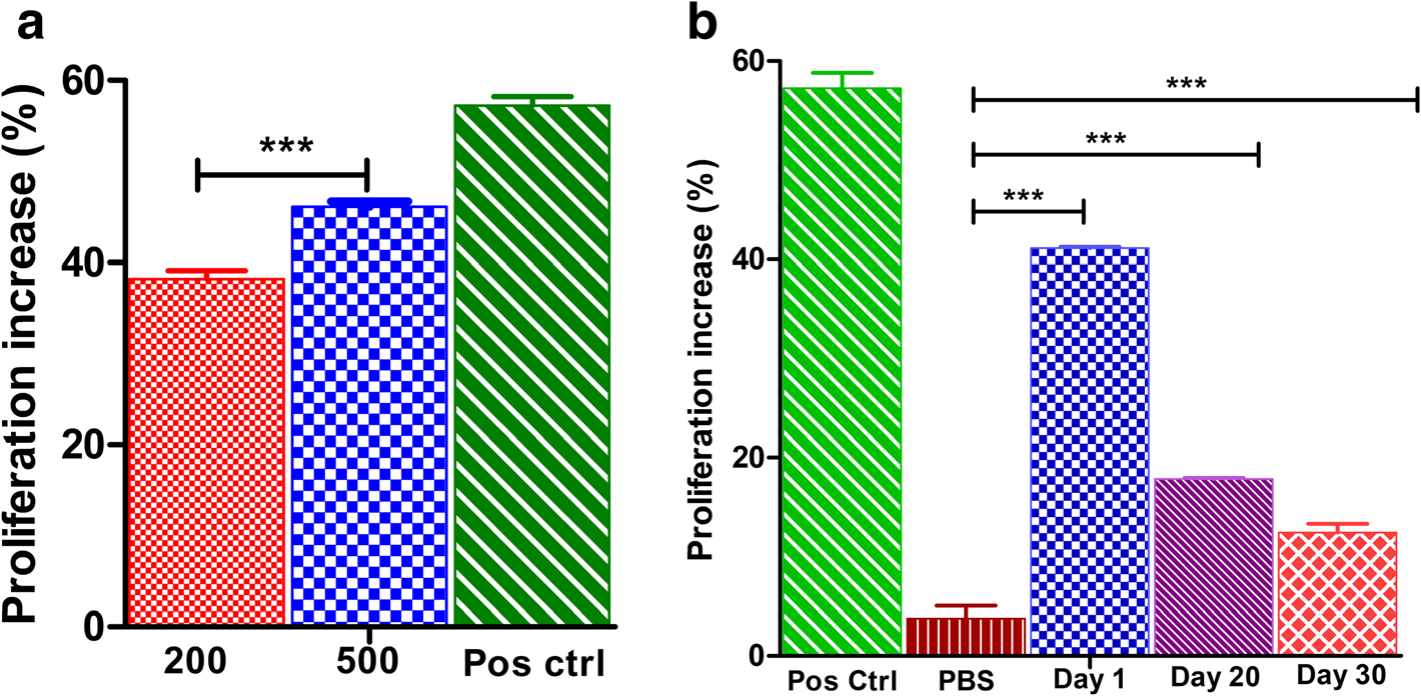
**a** MTT cell proliferation assay on human endometrium stromal cells (HESC) on 200 and 500 ng concentration of recombinant VEGF_121_. Absorbance read at 570 nm; data represented as mean ± S.D. from 3 replicates. Asterisks (***) indicate significant differences of *p* < 0.001 in comparison with 200 ng vs. 500 ng. **b** MTT cell proliferation assay on human endometrium stromal cells (HESC) on PLA encapsulated rVEGF_121_. Cells incubated with different concentrations for 24 h and compared with controls. Absorbance read at 570 nm; data represented as mean ± S.D. from 3 replicates. *Y*-axis showing the proliferation increase; asterisks (***) indicate significant differences of *p* < 0.001 in comparison with PBS (negative control) vs. day 1, day 20, and day 30

The proliferative efficacy of VEGF and VEGF-PLA particles were assessed in vitro in human endometrium stromal cell lines (HESC). Two hundred and 500 ng of VEGF_121_ and VEGF-PLA particles collected at different time points (days 1, 20, and 30) were tested for its effects in HESC (Fig. 2a). We found that recombinant VEGF_121_ and VEGF-PLA particles have proliferated the cells in vitro significantly (*p* < 0.0001)*.* Proliferation % of HESC cells was 38.22% in 200 ng of rVEGF_121_ and 46.12% in the cells induced with 500 ng of r VEGF_121_, while 57.26% increase was observed in the positive control. Cells treated with VEGF-PLA microparticles at different time points (days 1, 20, 30) showed significant (*p* < 0.0001) proliferative effects in HESC (Fig. 2b) such as 41.12%, 17.86%, and 12.45% respectively when compared with PBS-loaded PLA microparticles (3.80%).

### VEGF- and VEGF-loaded PLA particles are involved in the promotion of endometrium stromal cells migration

**Fig. 3:**
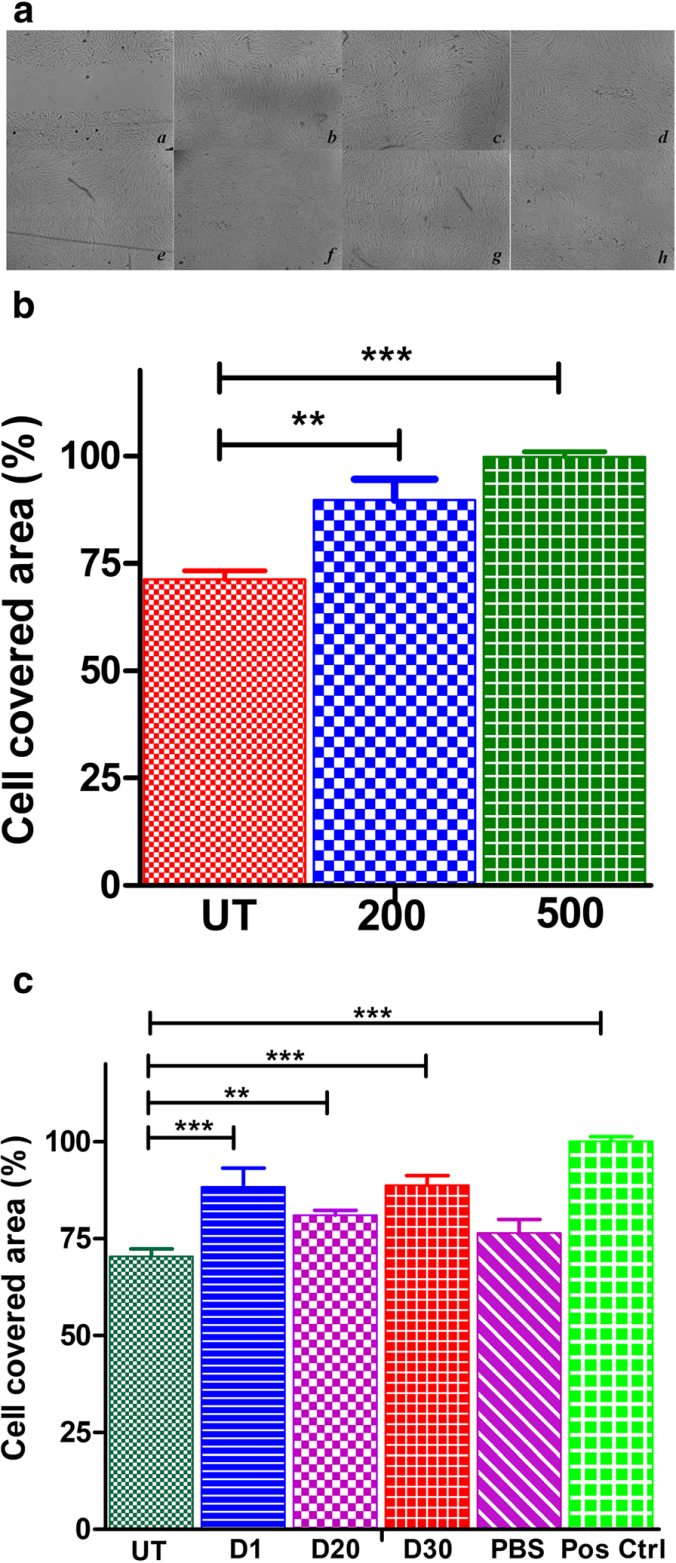
Effects of VEGF and VEGF-PLA on HESC migration: VEGF and VEGF-PLA stimulated HESC migration. HESC was grown to confluent and the monolayers were scratched with a pipette tip and stimulated with rVEGF_121_, 200 ng and 500 ng concentrations, and 1, 20, and 30 days’ release of VEGF-PLA; the cells were grown for 12 h and 24 h respectively. **A** Single picture representation of each group is shown; wound closure images was measured using Image J software. Images of the gap area induced at 12 h and 24 h: (a) untreated cells at 12 h; (b) 200 ng of rVEGF_121_ at 12 h; (c) 500 ng of rVEGF_121_ at 12 h; (d) positive control; (e) PLA–VEGF microparticles on day 1 at 24 h; (f) PLA–VEGF microparticles on day 20 at 24 h; (g) PLA–VEGF microparticles on day 30 at 24 h; (h) negative control. **B** The histogram showing the percentage of cell-covered area for different concentration of rVEGF_121_; asterisk indicates significant differences *p* < 0.001, *p* < 0.05. Cell covered percentage were compared with the untreated control (UT). Data represented (mean ± S.D., *n* = 3) at 12 h. Statistical significance shown is 200 ng and 500 ng of rVEGF_121_ vs. untreated group. **C** The histogram shows the percentage of cell-covered area at 24 h treated with rVEGF_121_-loaded microparticles (PLA-VEGF) collected on different days. Data represented as mean ± S.D., *n* = 3. Statistical significance (*p* < 0.001, *p* < 0.05) compared with the untreated control (UT)

The chemotactic potential of HESC was estimated through wound healing scratch assay (Fig. 3A). On treating HESC cells with rVEGF_121_ at 200 ng and 500 ng concentration, the cell-covered area was measured as 89.71% and 99.89% while the untreated cells showed 71.32% cell coverage (Fig. 3B). Cells on treatment with VEGF-PLA microparticles at different time points (days 1, 20, 30) showed significant migratory effect such as 88.24%, 80.96%, and 88.69% (days 1, 20, and 30), while 100% cell coverage in positive control and 70.46% in case of untreated control and 76.44% in PBS-loaded PLA particles (Fig. 3C).

### VEGF- and VEGF-loaded PLA particles induced PKBa/PIK3cB phosphorylation in endometrium stromal cells

**Fig. 4:**
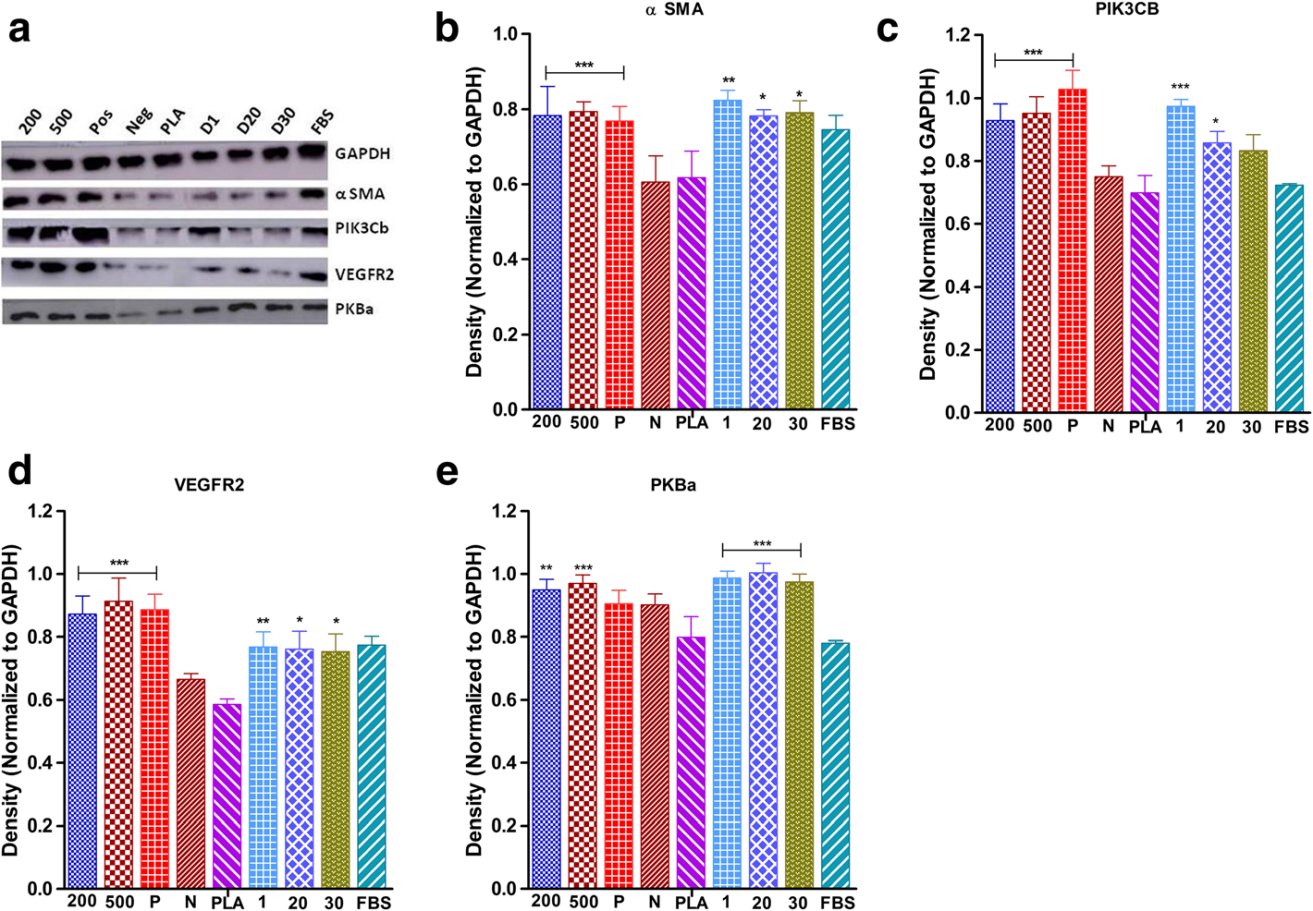
Effects of VEGF and PLA-VEGF on protein expression of α SMA, PIK3Cb, VEGFR2, and PKBa in HESC. **a** Western blotting images showing the levels of protein expression after stimulation with different concentrations of rVEGF_121_ and PLA-VEGF microparticles release. **b**–**e** Expression levels of α SMA (**b**), PIK3Cb (**c**), VEGFR2 (**d**), and PKBa (**e**) in HESC; the expression levels normalized with GAPDH expression. Data represented as mean ± S.D., *n* = 3. Statistical significance (*p* < 0.05, *p* < 0.01, *p* < 0.001) compared (200 and 500 ng of rVEGF_121_ with the positive control, while days 1, 20, and 30 with PLA-PBS (negative control))

VEGF- and VEGF-loaded PLA particles promoted cell migration in human endometrium stromal cells. To investigate whether PKBa, PIK3cB (signaling kinases), α SMA and VEGFR2 participated in migration; Western blot analysis was performed after treatment with rVEGF (200 and 500 ng) and VEGF-loaded PLA particles (days 1, 20, and 30) (Fig. 4a–e). The expression levels of PKBa, PIK3cB, α SMA, and VEGFR2 were markedly increased after the treatment with rVEGF- and VEGF-loaded PLA particles in three consecutive experimental groups in comparison to the control group. Similarly, alteration of PKBa, PIK3cB, α SMA, and VEGFR2 was observed in cells treated with VEGF at different concentrations, showing significantly elevated levels after VEGF stimulation. However, a low level of expression was observed in controls (PLA and cells without any stimulant). Results indicated that VEGF at different concentrations and VEGF released from PLA particles at different time points could activate the PKBa, PIK3cB, α SMA, and VEGFR2 pathways. The migrated cells on incubation with VEGF showed elevated levels of α SMA. VEGF thus induces smooth muscle-like differentiation in HESC which might regulate vascular lumen and angiogenesis in vivo.

## Discussion

Implantation is an important process for the development of an embryo; the implantation of an embryo depends on the thickness of the endometrium [14, 15]. It is believed that the thickness of the endometrium > 7 mm leads to a successful pregnancy [16, 17]. The female endometrium undergoes a routine cyclic process which requires proliferation and rapid arrest of neovascularization. During menstrual cycle, angiogenesis in the endometrium is controlled and regulated by several inhibitors and promoters which happen under the control of estradiol and progesterone [18]. Thromboplastin-1 is one of the important angiogenic inhibitors, stimulated by progesterone which is responsible for inhibiting excessive growth of the endometrial capillary system during the post-ovulatory phase of the menstrual cycle [19].

Presently, the mechanism of thin endometrium is unclear and no standardized treatment for the same has been elucidated. However, several studies are stating that vascularization of the endometrium is regulated by VEGF [20–23]. In a study conducted by Jing et al. [23], VEGF transfected with bone marrow-derived stromal cells (BMSCs) in a rat model showed that rats injected with VEGF-transfected BMSCs had thicker endometrium than the control group and BMSC group [23]. This method of transplantation also promoted the regeneration of endometrial cells and proven to be a stronger therapeutic effect. The most important promoters of endothelial growth are the vascular endothelial growth factor (VEGF) [24].

In this study, we showed the bioactivity of VEGF in human endometrium stromal cells; our previous studies [12] have proven the angiogenic potential and the activity of rVEGF and PLA-VEGF in human umbilical vein endothelial cells (HUVEC). Pro-angiogenesis is a process of regeneration and has been widely used in the regeneration of cardiovascular and endometrium. VEGF has been used in many clinical applications and therapeutics. We have reported in our earlier studies the wound healing properties of recombinant rVEGF_121_ and PLA microparticles in vitro in the HUVEC cell system [12].

VEGF significantly increases the adhesive properties and the outgrowth of the embryo in endometrial epithelial cells [25]. Endometrium thinning and damage is related to infertility and amenorrhea which affects women physically worldwide [26]. The decrease of blood flow leads to the damage of glandular epithelial cells and the inner membrane of the blood vessels; thus, the levels of VEGF expression also decrease leading to poor vascular growth and poor blood flow to the endometrium [27]. This proves that upregulating VEGF expression levels can thicken the endometrium and can induce the formation of blood vessels. Thus, VEGF-mediated signal pathway controls the endothelial cell proliferation and migration and helps in the promotion of angiogenesis. VEGF induces angiogenesis and is an important factor for endometrial growth. The effects of VEGF in endothelial cells and the angiogenic stimulations are mediated by PI3K pathway [17, 28]. The present study found that the protein expression levels of PKBa, PIK3cB, α SMA, and VEGFR2 improved after the treatment with rVEGF- and VEGF-loaded PLA particles than the controls. rVEGF_121_ and VEGF-PLA were able to proliferate and migrate in a better way than control groups, and the expression of PKBa, PIK3cB, and α SMA had elevated levels of protein levels after treatment of rVEGF_121_ and VEGF-PLA in HESC. It is proved that rVEGF_121_ and VEGF-PLA promote endometrium growth and have strong vascular regeneration effects. The high expression levels of α SMA confirm that the thickening of the endometrium is achieved because the smooth muscle cells are one of the important mediators in remodeling a tissue and help in angiogenesis [29]. Usually, in the process of wound healing, myofibroblasts continue to increase and they reduce the inflammation through the secretion of cytokines, chemokines, and protease inhibitors [30]. The fibroblasts increase expression of α SMA, transforming smooth muscle cells into myofibroblasts which help the wound to contract [31–33]. The upregulation of PI3K and AKT confirms the regeneration, and repair mechanisms are mediated through the PI3K/AKT signaling pathway and lead to angiogenesis. Therefore, it is understood that VEGF and VEGF-PLA activate PI3K/AKT signaling pathway through upregulation of the VEGF receptors (VEGFR2) which might have induced the repair in HESC.

VEGF and VEGF-PLA could promote the proliferation and migration and upregulate the levels of PKBa, PIK3cB, α SMA, and VEGFR2 in HESC. Li et al. designed a collagen scaffold with bFGF which increased endometrium regeneration in vivo [34, 35]. Lin et al. [36] loaded VEGF with collagen binding domain on the collagen scaffold which improved angiogenesis and regenerated the rat uterus. Their findings showed VEGF-collagen scaffold improved the pregnancy rate to 31.2% which was compared with VEGF injection alone. This study proves that VEGF released from the scaffold remodeled the extracellular matrix (ECM) and also activated matrix metalloprotease (MMP) [34, 36]. In another study conducted by Kim et al. [37], PLA loaded VEGF enhanced neo-blood vessel formation in rat subcutaneous model after 4 weeks of implantation of the scaffold. The above research shows that VEGF encapsulation to PLA can prevent direct exposure of the growth factor to endometrial cells. Thus, the encapsulated growth factor will be helpful to supply oxygen and nutrients to the target site and may be essential to activate the process of angiogenesis in a sustained controlled manner. However, VEGF and FGF have shown several promising results in formation of new blood vessels and angiogenesis; our interest lies in utilizing VEGF_121_, the most important isoform in endometrium for endometrium regeneration. However, VEGF is a potential candidate in stimulating angiogenesis and tissue regeneration; its half-life in its host environment is ~ 90 min [37–39]; therefore, to potentiate the effects of VEGF, we have encapsulated VEGF with PLA, which will supply VEGF for prolonged period in a sustained release mode to enrich the endometrium and regeneration. In this study, we have confirmed VEGF-A regulates angiogenesis by activating VEGFR-2 which is the major signal transducer in angiogenesis and their high expression can repair thin endometrium by promoting angiogenesis suggesting that VEGF and VEGF-PLA activate PI3K/AKT signaling pathway by upregulatingVEGFR2 levels.

## Conclusion

To conclude, this study is a simple approach for regenerating endometrium which accelerates wound healing in vitro. According to the results the comparison presented here are significant in comparison with the negative control. This means that VEGF and VEGF-PLA are capable of promoting angiogenesis in vitro. rVEGF121 and VEGF-PLA was able to proliferate and migrate in a better way than control groups and the expression of PKBa, PIK3cB, and α SMA had elevated levels of protein levels after treatment of rVEGF121 and VEGF-PLA in HESC. It is proved that rVEGF121 and VEGF-PLA promotes endometrium growth and has strong vascular regeneration effects. Thus, the combined effect could be used as a pro-angiogenic drug on further in vivo confirmation.

## Data Availability

Authors declare that all generated or analyzed data are included in the article.

## Abbreviations

μL: Microlitre
ABM: Applied Biological Materials
ANOVA: Analysis of variance
BCA: Bicinchoninic acid assay
BMSCs: Bone Marrow-Derived Stromal Cells
BSA: Bovine Serum Albumin
CO2: Carbon-di-oxide
DMEM: Dulbecco's Modified Eagle Medium
ECL: enhanced chemiluminescence
ECM: Extracellular matrix
ELISA: Enzyme-Linked Immunosorbent assay
FGF: Fibroblast Growth Factor
GAPDH: Glyceraldehyde 3-phosphate dehydrogenase
HESC: human immortalized endometrium stromal cell lines
HUVEC: Human Umbilical Vein Endothelial Cells
kDa: Kilo Dalton
mm: millimeter
MMP: matrix metalloprotease
MTT: 3-(4, 5-dimethylthiazol-2-yl)-2, 5-diphenyl tetrazolium br
ng: nanogram
NK: Natural killer
PBS: Phosphate Buffered Saline
PIK3Cb: Phosphoinositide-3-Kinase Catalytic Beta Polypeptide
PKBa: Protein Kinase B Alpha
PLA: poly-l-lactide
PVDF: Polyvinylidene fluoride
RIPA: Radio-immunoprecipitation assay buffer
Rpm: revolutions per minute
SDS-PAGE: Sodium Dodecyl Sulphate Polyacrylamide Gel Electrophoresis
TBST: Tris Buffered Saline-Tween 20
VEGF: Vascular Endothelial Growth Factor
VEGF: Vascular endothelial growth factor
VEGFR2: Vascular Endothelial Growth Factor Receptor 2
α SMA: alpha smooth muscle actin
